# Proximal junctional vertebral fracture-subluxation after adult spine deformity surgery. Does vertebral augmentation avoid this complication? A case report

**DOI:** 10.1186/1748-7161-7-16

**Published:** 2012-09-04

**Authors:** Nicomedes Fernández-Baíllo, José Miguel Sánchez Márquez, Francisco Javier Sánchez Pérez-Grueso, Alfredo García Fernández

**Affiliations:** 1 Orthopedic Surgery Department, Spine Service, La Paz University Hospital, Paseo de la Castellana, 261, 28046, Madrid, Spain

**Keywords:** Adult deformity, Proximal junctional fracture, Vertebroplasty

## Abstract

**Background:**

To report to the orthopedic community a case of vertebral fracture and adjacent vertebral subluxation through the upper instrumented vertebra after thoracolumbar fusion with augmentation of the cranial level.

**Methods:**

This report reviewed the patient`s medical record, her imaging studies and related literature. The possible factors contributing to this fracture are hypothesized.

**Results:**

A 70-year-old woman underwent decompressive surgery and posterolateral fusion for adult lumbar scoliosis. We used pedicular screws from T10 to S1 and iliac screw at the right side, augmented with cement at T10, T11, L1, L5 and S1; and prophylactic vertebroplasty at T9 to avoid the "topping-off syndrome".

Thirty days after discharge, without recognizable inciting trauma, the patient complained of pain in the lower thoracic area. The exam revealed overall neurological deficit below the level of fracture.

CT scan and MRI demonstrated a T10 vertebral collapse and T9 vertebral subluxation with morphologic features of flexion-distraction fracture through the upper edge of the screw.

At this point, the authors performed posterior decompression at T9 to T10 and extended posterolateral arthrodesis from T2 to T10.

To our knowledge, this is an unreported fracture.

**Conclusions:**

Augmentation of the cranial level in a long thoracolumbar fusion has been developed to avoid the junctional kyphosis and compression fractures at that level. We alert the orthopedic community that this augmentation may lead to further and more severe fractures, although this opinion requires investigation for confirmation.

## Background

Adjacent segment problems are well documented after spinal fusion. In osteoporotic patients with decreased bone strength and spinal fusion there is a higher risk of acute proximal collapse, due to the increased stiffness of the fused spinal segment that increases loads and motion within adjacent segments [1].

Several risk factors have been described for this “topping-off syndrome”: length of the fusion construct, reduced sagittal plane lordosis, female gender, age over 60 years and presence of osteoporosis [2].

Vertebral augmentation with cement (vertebroplasty or kyphoplasty) is a percutaneous procedure performed to stabilize vertebral insufficiency fractures and increase the mechanical strength of the fractured vertebral bodies. This procedure produces significant pain relief with limited complications in most patients undergoing this treatment for osteoporotic vertebral compression fractures [3,4]. However, this procedure has a well-known complication: the increased risk of adjacent vertebrae compression fractures. Nevertheless percutaneous vertebral augmentation has a role in the prevention of further fractures of the spine adjacent to a multilevel lumbar fusion.

We are aware of only two studies [1,5] assessing the value of vertebral augmentation as a prophylactic tool in the elderly or osteoporotic patients undergoing extended lumbar spine fusion; Hart et al. [1] hypothesized that routine prophylactic vertebral augmentation is cost-effective in patients older than 60 years undergoing extended lumbar fusions ending craneally within the thoracolumbar junction in comparison to the costs of revision surgery for patients suffering from proximal junctional acute collapse cranial to a multi-level lumbar fusion.

Watanabe et al. [6] describe two groups of adult patients with proximal vertebral fractures following spinal deformity surgery using segmental pedicle screw instrumentation without cement augmentation: upper instrumented vertebral collapse + adjacent vertebral subluxation and those with supra-adjacent vertebral fracture alone. The first group presented a shorter interval between the initial surgery and the fracture, hypokyphosis in the thoracic area before primary surgery and 40% had a severe neurologic deficit. Those authors proposed several risk factors for proximal junctional fracture: old age, osteopenia, severe global imbalance and marked correction of sagittal malalignment [6].

In this paper, we report a case of vertebral collapse at the upper instrumented level with adjacent vertebral subluxation after thoracolumbar fusion with augmentation at both levels, hypothesize about the possibility of an increased risk of this complication due to the effects of the vertebral augmentation and alert the orthopedic community about this phenomenon.

## Case presentation

**Figure 1:**
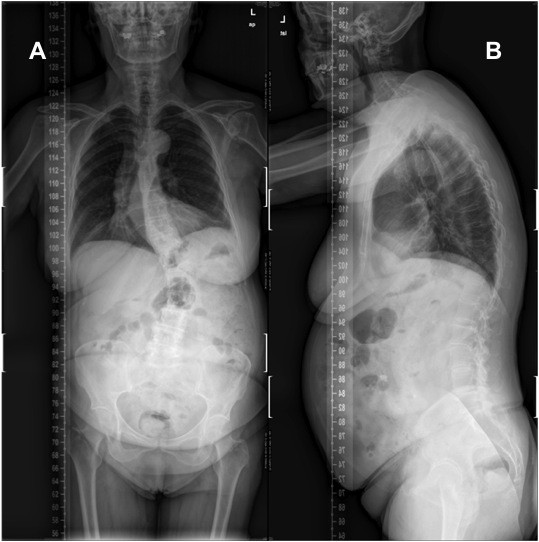
Preoperative p-a (A) and lateral (B) x-rays demonstrating degenerative lumbar scoliosis with anterolateral lystesis at L2-L3-L4 and L5.

**Figure 2:**
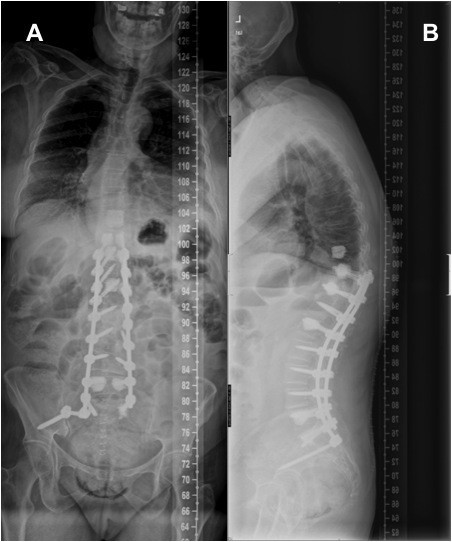
**Postoperative p-a and lateral x-rays showing a posterolateral arthrodesis from T10 to S1 and iliac screw in the right side, augmented with cement at T10, T11, L1, L5 and S1.** Prophylactic vertebroplasty at T9 was performed to avoid the “topping-off syndrome”. Balanced profile in both frontal and sagittal planes were obtained.

A 70-year-old woman with degenerative lumbar scoliosis suffering severe low back pain and neurogenic claudication, aggravated during ambulation, underwent decompressive laminectomy at L4-L5 and posterolateral fusion with a reasonably good result. Four years later, her condition had worsened including neurogenic claudication and low back pain. Her primary care doctor referred her to our service as a new patient. At this point, the x-ray (Figure 1) and MRI showed a left lumbar curve with a 34ª Cobb angle between T11 and L4, anterolateral lysthesis at L2-L3-L4-L5 and central and subarticular lateral recess stenosis. We used titanium 5.5 mm fenestrated pedicular screws (Expedium, DePuy Spine, Raynham, MA, USA) on both sides at each level from T10 to S1 and an iliac screw in the right side (Figure 2), augmented with cement at T10, T11, L1, L5 and S1 (Confidence Spinal Cement System, DePuy Spine, Raynham, MA, USA) and prophylactic vertebroplasty at T9 to avoid the “topping-off syndrome”. There were no intraoperative pedicular fractures and special care was taken to preserve the structures of the tension band (posterior ligaments, facet joints, multifidi muscles) in the segment above the instrumentation.

The patient had a torpid postoperative recovery, complaining of pain in the thoracolumbar area, but her ability to perform different physical activities increased daily. The x-ray on day 3 post-surgery showed no abnormal finding. She was discharged walking 8 days after surgery.

One month after discharge, without recognizable inciting trauma, the patient complained of increasing spontaneous pain in the lower thoracic area and neurological impairment in the lower limbs. Physical examination revealed tenderness in the lower thoracic spine without a palpable defect between the posterior spinous processes. Neurological examination was abnormal without associated injuries. The motor exam revealed an overall decrease in lower limbs muscle strength (2/5 on the left leg, and 3/5 on the right leg) with inability to walk, and response in the patellar and Achilles reflexes was increased, Babinsky reflex was positive on both sides and abnormal bladder and bowel function were reported.

**Figure 3:**
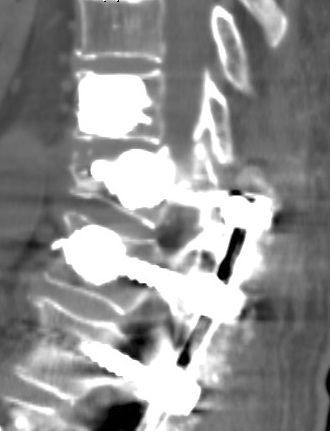
Computed tomography revealing a collapse and wedging of the T10 vertebral body and distraction pattern fracture at pedicles through the upper edge of the screw, with anterior subluxation of T9.

**Figure 4:**
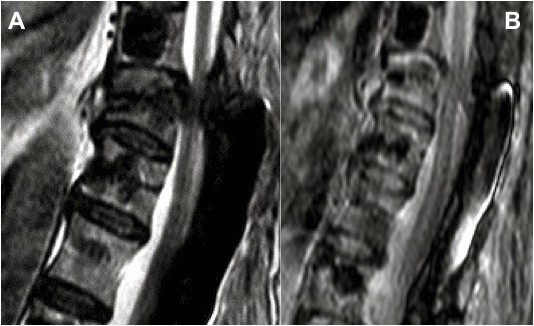
Two sagittal MRI views showing rupture of posterior elements at T9-T10 with high signal intensity and abnormal intracanal tissue compressing the spinal cord.

The initial supine radiographs showed a slight junctional kyphosis but no indications of vertebral fracture. CT scan (Figure 3) and MRI (Figure 4) demonstrated a collapse and wedging of the T10 vertebral body and a distraction pattern injury at the upper edge of the screw in the T10 pedicles, rupture of the posterior elements at T9-T10 with a high signal intensity consistent with hemorrhage and edema and subluxation at the cranial level. Abnormal intracanal tissue was noted compressing the spinal cord.

**Figure 5:**
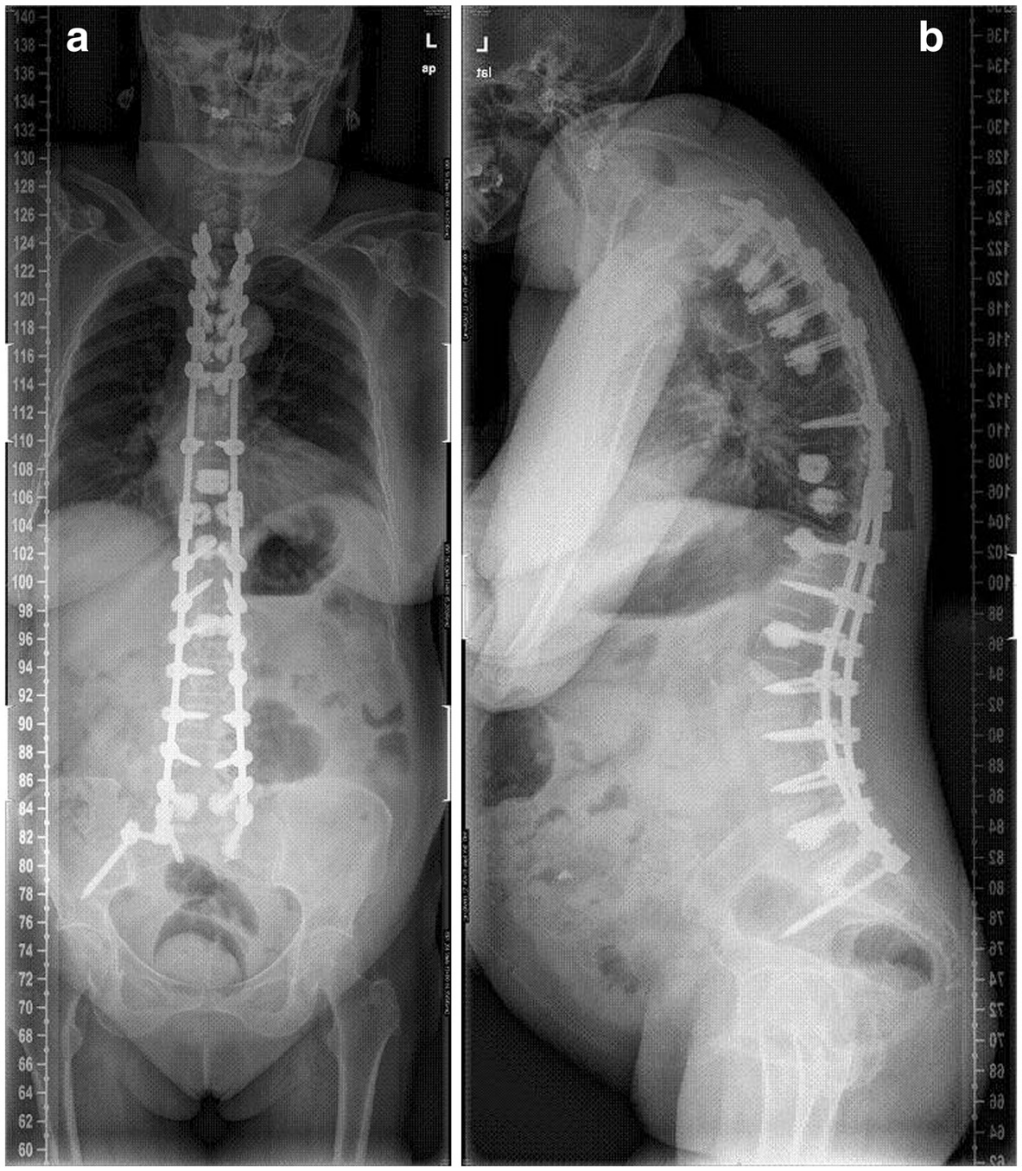
**Post-revision surgery p-a (a) and lateral (b) x-ray.** The fusion was extended to T2 with decompression at T9-T10, using bilateral pedicular screws augmented with cement at T2, T3, T4, T5 and T6. Satisfactory profile in both frontal and sagittal planes were obtained.

With the confirmation of a T10 vertebral compression fracture with subluxation of the adjacent level and neurological impairment, the authors performed posterior decompression at T9-T10 and extended posterolateral arthrodesis (Figure 5) from T2 to T10 using bilateral pedicular screws augmented with cement at T2,T3,T4,T5 and T6, and non-cemented screws at T8 (Expedium-Confidence Spinal Cement System, DePuy Spine, Raynham, MA, USA). Samples of the intracanal tissue were obtained for histological study, which reported a ring of fibrous and vascular tissue compatible with soft fracture repairing callus.

The patient had prompt improvement in back pain and was mobilized routinely. At 3 months follow-up, she was able to walk without pain and the neurological exploration was normal.

## Conclusions

A number of previous studies have reported perioperative and postoperative complication rates in adult spinal deformity surgery of up to or more than 40% [7]. One major complication is a compression fracture of the last instrumented and/or the supra-adjacent vertebral body [7], this requires reintervention and extension of spinal fusion.

Augmentation of pedicle screws with PMMA or calcium phosphate cement has been shown to improve the initial fixation and fatigue strength of instrumentation in osteoporotic vertebrae, and it also decreases the likelihood of compression fracture at the same level [8]. Additional vertebroplasty of the neighboring 1 to 2 uninstrumented levels has been applied attempting to avoid the junctional kyphosis and compression fractures of cranial vertebrae.

As mentioned before, Watanabe et al. [6] described two patterns of proximal vertebral fracture following spinal deformity surgery in adults receiving segmental pedicle screw instrumentation: upper instrumented vertebral collapse + adjacent vertebral subluxation and supra-adjacent vertebral fracture alone. In an attempt to avoid these complications in our patient, we augmented the upper instrumented vertebra and its adjacent level, but this created excessive distractive stress in the posterior element, resulting in a compression fracture of the upper instrumented vertebral body (even after being augmented with cement) with distraction of posterior elements and pedicles through the upper edge of the screw and subluxation of the adjacent level.

We hypothesize that vertebral augmentation could increase the risk of this complicated fracture. There are several factors that might lead to this complication:

Incomplete cement filling of the vertebra can create an area of weakness between two cemented areas (from the edge of the upper screws and the cranially-adjacent cemented vertebra) that behaves like an osteoporotic vertebra between two cemented vertebras.

The insertion of the pedicular screw leads to pedicle weakness.

Ending the instrumentation construct just below the apex of the postoperative thoracic kyphosis is too-frequently associated with junctional kyphosis/fractures [9-11].

The case presented here is concerning. The juxtaposition of these factors, partial vertebral cement filling, a positive sagittal balance and structurally-weakened pedicles may set the scene for a very early posterior element distraction failure combined with a compression fracture in the uncemented area.

Ending the construct too low, just below the apex of the kyphosis, can predispose to junctional kyphosis and compression fracture at the cranial level. According to the preoperative radiographs, the upper instrumented vertebra (T10) was located several levels below the apex (T8). In an attempt to improve the preoperative sagittal imbalance we surgically increased the lumbar lordosis (from 45ª preoperatively to 62ª in the postoperative x-ray), this may have led to a new sagittal profile and a greater kyphosis, moving the level of the apex. Attention should be paid to this point, because excessive bending of the rod in the lumbar spine can facilitate the migration of the thoracic kyphosis apex.

We think that the augmentation of the upper instrumented vertebra and the supra-adjacent level, in an attempt to avoid the complications described by Watanabe et al. [6], does not solve the problem and may lead to further and more severe fractures, like the one described in this paper, although this opinion requires biomechanical and/or clinical investigation for confirmation.

Our service has used this technique successfully in more than 15 patients in the last two years. To date, this is the only occurrence of this complication. When successful, vertebral augmentation of the cranial level in a long thoracolumbar fusion solves the topping off syndrome and avoids major disabilities.

This is a single case report and does not completely explain high incidence of complications in the junctional level after surgery for adult spinal deformity. Our purpose is to alert the orthopedic community to this phenomenon.

We would advise that any patient with a long thoracolumbar fusion with cranial level augmentation be closely monitored.

## Consent

Written informed consent was obtained from the patient for publication of this Case Report and any accompanying images. A copy of the written consent is available for review by the Editor-in-Chief of this journal.

## Competing interests

The authors of this paper do not have any conflict of interest, financial or non-financial, related with the study.

## Authors’ contribution

NFB and FJSPG performed the first surgery. NFB, AGF and JMSM performed the second surgery. All authors participated in the design and redaction of the text and conclusions, and posterior corrections. All authors read and approved the final manuscript.

## References

[B1] Hart RA Prendergast MA Roberts WG Proximal junctional acute collapse cranial to multi-level lumbar fusion: a cost analysis of prophylactic vertebral augmentation Spine J 2008 8 875 881 10.1016/j.spinee.2008.01.015 18375188

[B2] Cahill DW Etebar S Risk factors for adjacent segment failure following lumbar fixation with rigid instrumentation for degenerative instability J Neurosurg 1999 90 163 9 10199244 10.3171/spi.1999.90.2.0163

[B3] Maid ME Farley S Holt RT Preliminary outcomes and efficacy of the first 360 consecutive kyphoplasties for the treatment of painful osteoporotic vertebral compression fractures Spine J 2005 5 244 55 10.1016/j.spinee.2004.09.013 15863078

[B4] Lee MJ Dumonski M Cahill P Percutaneous treatment of vertebral compression fractures: a meta-analysis of complications Spine 2009 34 1228 32 10.1097/BRS.0b013e3181a3c742 19444071

[B5] Lattig F Bone cement augmentation in the prevention of adjacent segment failure after multilevel adult deformity fusion J Spinal Disord Tech 2009 22 439 443 10.1097/BSD.0b013e31818d6493 19652572

[B6] Watanabe K Lenke LG Bridwell KH Proximal junctional vertebral fracture in adults after spinal deformity surgery using pedicular screw constructs Spine 2010 35 138 145 10.1097/BRS.0b013e3181c8f35d 20081508

[B7] Bradford DS Tay BK Hu SS Adult scoliosis: surgical indications, operative management, complications and outcomes Spine 1999 24 2617 2639 10.1097/00007632-199912150-00009 10635525

[B8] DeWald CJ Stanley T Instrumentation-related complications of multilevel fusions for adult spinal deformity patients over age 65: surgical considerations and treatment options in patients with poor bone quality Spine 2006 31 19 suppl 144 151 10.1097/01.brs.0000236893.65878.39 16946632

[B9] Kim YJ Bridwell KH Lenke LG Sagittal thoracic decompensation (SThD) following adult lumbar spinal instrumentation and fusion to L5 or S1: causes, incidence, and risk factor analysis Spine 2006 31 2359 66 10.1097/01.brs.0000238969.59928.73 16985465

[B10] Swank ML Adjacent segment failure above lumbosacral fusions instrumented to L1 or L2. Podium presentation at the Scoliosis Research Society 37th annual meeting 2002 September 18-21; Seattle, WA. USA

[B11] Suk SI Kim JH Lee SM Incidence of proximal adjacent failure in adult lumbar deformity correction. Podium presentation at the Scoliosis Research Society 38th annual meeting 2003 September 10-13; Quebec City, Canada

